# Social support as a health resource in Germany

**DOI:** 10.17886/RKI-GBE-2017-131

**Published:** 2017-12-13

**Authors:** Lea-Sophie Borgmann, Petra Rattay, Thomas Lampert

**Affiliations:** Robert Koch Institute, Department of Epidemiology and Health Monitoring, Berlin

**Keywords:** SOCIAL SUPPORT, RESOURCES, SOCIAL FACTORS, HEALTH MONITORING, GERMANY

## Abstract

Social support is a psychosocial resource that results from social ties and networks. It has a significant impact on health and can improve mental well-being, reduce stress and mitigate the impact of unfavourable living conditions. The GEDA 2014/2015-EHIS survey undertaken by the Robert Koch Institute (RKI) is used to examine the extent and distribution of perceived social support among the adult population in Germany (n=23,617). The results show that both women and men largely feel supported by the people they are close to and their neighbours. However, perceived social support is not distributed equally across the population: the elderly, people with low levels of education and the unemployed report relatively frequently that they receive low levels of social support. As such, the sections of the population that are more frequently affected by health problems are also less likely to be able to rely on social support.

## Introduction

Social support arises out of factors such as social ties and networks and is an important resource for health. Social support can have different, sometimes overlapping functions. On the one hand, it includes emotional support, which is characterised by feelings of affection and caring, and a perception of being understood by others. On the other, social support also involves instrumental support such as in the help people receive in dealing with specific everyday tasks including through the financial support or by undertaking unpaid work. It can also include help with shopping or paying bills, assistance with decision-making, opinions about the decisions a person has taken, and the provision of informational support [1, 2]. Data can be gathered on social support for research in various ways, but is mainly done by collecting data on perceived or expected social support, as well as the actual social support that a person receives [3].

It has been demonstrated empirically that perceived social support can have an impact on various aspects of health [2, 4-7]. Links between perceptions of psychological well-being, self-confidence and self-efficacy, but also to health-related behaviour such as physical exercise, tobacco and alcohol consumption and perceived social support have been observed [1]. People who do not feel that they are receiving sufficient social support are more frequently burdened by chronic stress, more likely to suffer from physical ailments and illnesses, and have a higher risk of mortality [1, 2, 8, 9]. Moreover, research on the adult population in Germany has shown that low levels of perceived social support among women and men in all age groups are associated with a significant increase in the prevalence of depression [10].

**GEDA 2014/2015-EHIS**

**Data holder:** Robert Koch Institute

**Aims:** To provide reliable information about the population’s health status, health-related behaviour and health care in Germany, with the possibility of a European comparison

**Method:** Questionnaires completed on paper or online

**Population:** People aged 18 years and above with permanent residency in Germany

**Sampling:** Registry office sample; randomly selected individuals from 301 communities in Germany were invited to participate

**Participants:** 24,016 people (13,144 women; 10,872 men)

**Response rate:** 26.9%

**Study period:** November 2014 - July 2015

**Data protection:** This study was undertaken in strict accordance with the data protection regulations set out in the German Federal Data Protection Act and was approved by the German Federal Commissioner for Data Protection and Freedom of Information. Participation in the study was voluntary. The participants were fully informed about the study’s aims and content and about data protection. All participants provided written informed consent.

More information in German is available at www.geda-studie.de

## Indicator

Data was gathered as part of the GEDA 2014/2015-EHIS study through information provided by respondents as part of a paper or online questionnaire. The level of perceived social support was assessed using the Oslo 3-Item Social Support Scale (Oslo-3 Scale) [11]. The questionnaire included the following questions on perceived social support: ‘How many people are so close to you that you can count on them if you have serious problems?’ (possible answers were: ‘none’, ‘1 or 2’, ‘3 to 5’ and ‘6 or more’); ‘How much concern do people show in what you are doing?’ (possible answers were: ‘a lot of concern and interest’, ‘some concern and interest’, ‘uncertain’, ‘little concern and interest’ and ‘no concern and interest’); and ‘How easy can you get practical help from neighbours if you should need it?’ (possible answers were: ‘very easy’, ‘easy’, ‘possible’, ‘difficult’, ‘very difficult’). The answers provided were developed into a scale used to measure people’s perceived social support. This was done by adding the single-point values gained from the three questions to form an index; possible values ranged from 3 to 14 points. Results ranging from 3 to 8 points were classified as a low level of support, 9 to 11 points as a medium level of support, and 12 to 14 points as indicating a high level of social support [12].

The analyses undertaken for the GEDA study are based on the information provided by 23,617 participants aged over 18 (12,921 women; 10,696 men) with valid data on social support. Analyses of (self-defined) employment status were limited to people of working age (up to 64 years). The calculations were carried out using a weighting factor that corrected the sample for deviations from the German population structure (as of 31 December 2014) in terms of gender, age, municipality type and level of education. ‘Municipality type’ refers to the degree of urbanisation in a particular area and corresponds to the regional distribution in Germany. The International Standard Classification of Education (ISCED) was used to classify the information on education provided by the participants [13]. A statistically significant difference between groups is assumed to have been demonstrated when confidence intervals do not overlap.

A detailed description of the methodology used for GEDA 2014/2015-EHIS can be found in Lange et al. 2017 [14] as well as in the article German Health Update: New data for Germany and Europe in issue 1/2017 of the Journal of Health Monitoring.

## Results and discussion

In Germany, 29.2% of women and 25.4% of men report that they experience high levels of social support (Table 1 and Table 2). A further 53.2% of women and 55.7% of men report to perceive medium levels of social support. Only 17.6% of women and 19.0% of men stated that they perceived low levels of social support. The proportion of women who perceive low levels of social support increases slightly with age. However, the differences that were identified in terms of age and gender are not statistically significant.

The data demonstrate that the degree of social support that a person perceives is linked to their level of education: the proportion of women and men with low levels of social support is lowest among people with high levels of education. This difference is significant among women for all respondents aged up to the age of 64. The same can be said of men aged over 30. In terms of the 30- to 64-year olds, high levels of social support are particularly frequently seen among women with high levels of education (when compared with the group with the lowest level of education). Among men, this applies to 30- to 44-year-olds.

**Figure 1:**
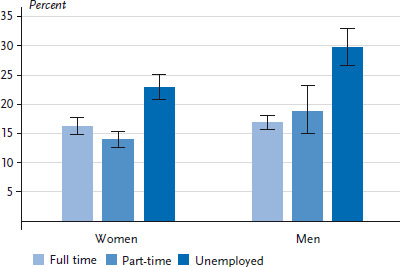
Proportion of low levels of perceived social support (Oslo 3-Item Social Support Scale) by gender and employment (n=10,140 women; n=7,847 men) Source: GEDA 2014/2015-EHIS

With regard to employment status, the share of low levels of social support among unemployed women and men aged between 18 and 64 years was significantly higher than among full-time and part-time employees (Figure 1). Among both genders, the proportion of unemployed people who perceived low levels of social support was particularly high in middle age (people aged between 30 and 64). High levels of social support are seen significantly more frequently among men aged 30 or above in full-time employment than among unemployed men in the same age group. This also applies to women aged between 45 and 64 years (data not shown).

The results of the GEDA 2014/2015-EHIS study show that more than four fifths of the adult population in Germany perceive themselves as having social ties and networks that provide them with medium to high levels of social support. However, women and men with low levels of education are more likely to perceive themselves as receiving low levels of social support from their social environment. In addition, unemployed women and men, in particular, report that they perceive low levels of social support. Therefore, the population groups that are more frequently affected by health problems are also less likely to be able to rely on social support. These results are consistent with the data gathered for previous GEDA studies as well as a study on the ties between social support and health conducted within data of the Socio-Economic Panel [4, 6].

## Key statements

- More than a quarter of adults in Germany state that they receive a high level of social support.
- 17.6% of women and 19.0% of men state that they receive very little social support.
- Adults with lower levels of education are more likely to report low levels of perceived social support than adults with high levels of education.
- The proportion of people who state that they perceive low levels of social support is significantly higher among adults who are unemployed.

## Figures and Tables

**Table 1:** Social support (Oslo 3-Item Social Support Scale) for women by age and education (n=12,921 women; n=10,696 men) Source: GEDA 2014/2015-EHIS

Women	Low support		Medium support		Strong support	
	%	(95% CI)	%	(95% CI)	%	(95% CI)
**Women (total)**	**17.6**	**(16.6-18.6)**	**53.2**	**(52.1-54.3)**	**29.2**	**(28.1-30.3)**
Low education	23.0	(21.0-25.2)	51.7	(49.4-54.0)	25.2	(23.1-27.5)
Medium education	17.0	(15.8-18.2)	54.2	(52.9-55.6)	28.8	(27.4-30.2)
High education	12.3	(11.0-13.7)	51.6	(49.8-53.4)	36.1	(34.3-37.9)
**18-29 Years**	16.7	(14.5-19.2)	55.1	(52.5-57.6)	28.2	(25.9-30.5)
Low education	23.4	(18.2-29.5)	53.2	(47.0-59.3)	23.4	(18.5-29.1)
Medium education	16.1	(13.7-18.9)	54.9	(51.8-58.0)	29.0	(26.0-32.1)
High education	9.5	(6.9-13.1)	57.9	(53.3-62.4)	32.6	(28.6-36.9)
**30-44 Years**	17.9	(16.1-19.7)	52.5	(50.0-55.1)	29.6	(27.3-32.0)
Low education	29.3	(23.4-36.1)	45.8	(38.2-53.5)	24.9	(18.8-32.2)
Medium education	17.7	(15.5-20.0)	54.9	(51.8-58.0)	27.4	(24.6-30.4)
High education	12.2	(10.1-14.7)	50.4	(46.6-54.1)	37.5	(33.9-41.2)
**45-64 Years**	15.9	(14.6-17.3)	53.4	(51.7-55.0)	30.7	(29.1-32.4)
Low education	20.5	(17.1-24.4)	55.4	(50.9-59.7)	24.1	(20.6-28.0)
Medium education	15.9	(14.3-17.7)	53.4	(51.2-55.7)	30.7	(28.6-32.8)
High education	11.7	(9.9-13.7)	51.4	(48.4-54.4)	36.9	(34.1-39.8)
**≥65 Years**	20.2	(18.3-22.3)	52.3	(49.9-54.7)	27.4	(25.3-29.6)
Low education	22.6	(19.7-25.7)	50.8	(47.0-54.5)	26.7	(23.3-30.3)
Medium education	18.8	(16.2-21.6)	54.5	(51.3-57.7)	26.8	(24.0-29.7)
High education	17.0	(13.8-20.8)	48.5	(43.5-53.5)	34.5	(29.9-39.4)
**Total (women and men)**	**18.3**	**(17.6-19.0)**	**54.4**	**(53.6-55.2)**	**27.3**	**(26.6-28.1)**

CI=confidence interval

**Table 2:** Social support (Oslo 3-Item Social Support Scale) for men by age and education (n=12,921 women; n=10,696 men) Source: GEDA 2014/2015-EHIS

Men	Low support		Medium support		Strong support	
	%	(95% CI)	%	(95% CI)	%	(95% CI)
**Men (total)**	**19.0**	**(18.0-20.0)**	**55.7**	**(54.5-56.8)**	**25.4**	**(24.4-26.4)**
Low education	26.2	(23.4-29.2)	52.6	(49.3-55.9)	21.2	(18.5-24.2)
Medium education	19.4	(18.1-20.7)	56.0	(54.4-57.6)	24.6	(23.2-26.1)
High education	14.1	(12.9-15.4)	56.8	(55.1-58.5)	29.1	(27.6-30.6)
**18-29 Years**	18.9	(16.5-21.6)	55.0	(52.0-57.9)	26.1	(23.5-28.9)
Low education	23.4	(18.0-29.9)	51.0	(44.3-57.6)	25.6	(20.0-32.1)
Medium education	17.7	(14.8-21.0)	57.4	(53.3-61.4)	24.9	(21.7-28.4)
High education	15.2	(10.8-21.0)	51.8	(46.1-57.4)	33.1	(27.6-39.0)
**30-44 Years**	19.2	(17.1-21.4)	55.6	(52.9-58.2)	25.3	(23.2-27.4)
Low education	34.4	(27.0-42.7)	49.9	(42.0-57.9)	15.6	(10.3-23.1)
Medium education	18.5	(15.8-21.6)	58.0	(54.2-61.6)	23.5	(20.7-26.6)
High education	13.3	(10.7-16.4)	54.3	(50.5-58.0)	32.4	(29.2-35.8)
**45-64 Years**	18.9	(17.5-20.4)	55.9	(53.9-57.8)	25.2	(23.7-26.8)
Low education	24.4	(20.0-29.5)	54.0	(48.1-59.8)	21.5	(17.5-26.3)
Medium education	20.6	(18.5-22.9)	54.8	(52.0-57.6)	24.6	(22.3-27.0)
High education	13.7	(12.1-15.6)	58.5	(56.0-60.9)	27.8	(25.6-30.1)
**≥65 Years**	19.0	(17.2-20.9)	56.0	(53.7-58.2)	25.1	(23.0-27.3)
Low education	24.8	(20.3-29.9)	55.9	(49.9-61.6)	19.3	(15.4-23.9)
Medium education	19.6	(16.8-22.7)	54.7	(51.4-58.0)	25.7	(22.6-29.1)
High education	15.2	(13.1-17.5)	58.5	(55.2-61.8)	26.3	(23.4-29.5)
**Total (women and men)**	**18.3**	**(17.6-19.0)**	**54.4**	**(53.6-55.2)**	**27.3**	**(26.6-28.1)**

CI=confidence interval
